# General psychiatric symptoms among Bangladeshi people approximately one year after the onset of the COVID-19 pandemic

**DOI:** 10.1186/s12888-022-04232-3

**Published:** 2022-09-19

**Authors:** Rajon Banik, Md. Saiful Islam, Masruk Ahmed, Kamrun Nahar Koly, Mahfuza Mubarak, Mahmudur Rahman, Zu Wei Zhai, Md. Tajuddin Sikder, Marc N. Potenza

**Affiliations:** 1 Department of Public Health and Informatics, Jahangirnagar University, Savar, Dhaka, 1342 Bangladesh; 2 Centre for Advanced Research Excellence in Public Health, Savar, Dhaka 1342 Bangladesh; 3 Health System and Population Studies Division, International Centre for Diarrhoeal Disease Research, Bangladesh (icddr,b), Mohakhali, Dhaka, 1212 Bangladesh; 4 Program in Neuroscience, Middlebury College, Middlebury, VT USA; 5 Department of Psychiatry and Child Study Center, Yale School of Medicine, New Haven, CT USA; 6 Connecticut Mental Health Center, New Haven, CT USA; 7 Connecticut Council on Problem Gambling, Wethersfield, CT USA; 8 Department of Neuroscience, Yale University, New Haven, CT USA

**Keywords:** COVID-19, Loneliness, Anxiety, Depression, Insomnia, Fear, Bangladesh

## Abstract

**Background:**

Coronavirus disease-2019 (COVID-19) has had negative physical and mental impacts on people globally. The current study examined general psychiatric symptoms (fear, anxiety, depression, and insomnia) and loneliness, and their interrelationships and correlates among Bangladeshi individuals approximately 1 year after the onset of the COVID-19 outbreak.

**Methods:**

An internet-based cross-sectional survey was conducted among 1004 Bangladeshi people (51.8% male; mean age: 25.41 ± 7.80; age range: 18–60 years). Data were collected using a semi-structured e-questionnaire including informed consent, socio-demographics, lifestyle measures, and psychometric tools assessing loneliness, anxiety, depression, insomnia, and fear of COVID-19.

**Results:**

Sizeable participants screened positive for loneliness (63.5%), anxiety (26.3%), depression (46.4%), and insomnia (50.7%). Considerable numbers of respondents also reported fear of COVID-19. In hierarchical regression analyses, loneliness, anxiety, depression, insomnia, and fear of COVID-19 were associated with socio-demographic and lifestyle factors. Loneliness, anxiety, depression, insomnia, and fear of COVID-19 were positively correlated with each other (*p* < 0.001). In exploratory path analyses, anxiety, depression, and insomnia mediated the relationship between loneliness and fear of COVID-19.

**Conclusions:**

The findings indicate that many people in Bangladesh have experienced psychiatric concerns approximately 1 year after the onset of the COVID-19 outbreak. Investigation into empirically supported interventions and their implementation is needed.

**Supplementary Information:**

The online version contains supplementary material available at 10.1186/s12888-022-04232-3.

## Introduction

Worldwide, the outbreak of coronavirus disease-2019 (COVID-19) is a significant public health concern [1]. COVID-19 originated in Wuhan, Hubei Province, China in December 2019 [2, 3]. The COVID-19 pandemic represents a huge disease burden, infecting over 556 million people around the globe as of July 15, 2022 [4]. Symptoms range from asymptomatic infection to severe respiratory infection and mortality [5]. In addition to the physical health consequences, public health authorities globally have expressed concerns over an international mental health crisis due to quarantine, social isolation, financial strain, and the threat of infection [6, 7]. Experts remain uncertain regarding the differential trajectories of the COVID-19 pandemic across jurisdictions, projected numbers of cases and deaths, and to what extent restrictive measures may disrupt daily life [8]. The unpredictability and ambiguity surrounding COVID-19 may trigger numerous psychiatric and related phenomena, including loneliness, anxiety, depression, sleep concerns such as insomnia, and fear [8–15], in varying forms and to different degrees [16].

Major epidemics and pandemic outbreaks can have profound and wide-ranging psychological impacts on people [17]. Even people not directly affected by the disease may have considerable psychological problems stemming from the pandemic, as people may experience uncertainty and fear of falling sick or dying, feelings of helplessness, and stigma [18]. These experiences may impact their overall mental health, sometimes throughout their lives [19]. In the setting of physical separation, which mandates reducing in-person social interactions, rates of loneliness may rise, potentially increasing the prevalence of mood disorders, self-harm, and suicide, as well as exacerbating pre-existing mental health conditions [12]. The extent to which loneliness may operate to worsen mental health that may then impact abilities or tendencies to adapt to stressors like the COVID-19 pandemic warrants consideration.

Bangladesh has been facing tremendous challenges since the first verified COVID-19 outbreak on March 8, 2020 [20] with a total of over 1.9 million transmissions with a 14% infection rate as of July 15, 2022 [21]. To deal with this challenge, the government has imposed numerous pandemic-related limitations (e.g., lockdown, quarantine, and social isolation) to address this pandemic [22]. In this environment, individuals may feel isolated, potentially compromising their psychological well-being [11, 15, 22]. A prior study early in the pandemic reported that 33.7% of Bangladeshi adults suffered from anxiety, with 57.9%, and 59.7% experiencing depressive symptoms and stress, respectively [15]. Another Bangladeshi study found that 27.8% of participants were depressed, and depression was associated with COVID-19-related fear [23]. Sleep concerns were also prominent among Bangladeshi residents early during the COVID-19 pandemic, with about 2.8% of participants reporting severe forms of insomnia [14]. Recent publications in Bangladesh suggest factors such as age, gender, socioeconomic status, education level, occupation, and perceptions of COVID-19 relate to psychiatric disorders [15, 24]. Furthermore, fear of infection, financial uncertainty, inadequate food supply, and physical inactivity were related to anxiety and depression [25].

While prior studies of specific psychiatric concerns in Bangladesh have been reported during the initial period of the outbreak of COVID-19 [11, 15, 24, 26], little is known about the longer-term mental health impacts due to this ongoing pandemic. Bangladesh has been encountering a surge in COVID-19 infections, with nearly 50% more deaths every day than during the previous peak in June 2020 [27]. Given these circumstances, it is crucial to understand the current psychiatric concerns of Bangladeshi people as the pandemic progresses. The objective of this study was to investigate the prevalence and associated factors of psychiatric symptoms among Bangladeshi people approximately 1 year after the first COVID-19 diagnosis. Additionally, exploratory path analyses were conducted to examine the extent to which loneliness may operate through psychiatric symptoms (anxiety, depression, and insomnia) to impact fear of COVID-19.

## Methods

### Study design and setting

The study employed a cross-sectional design. Data were collected from January to March 2021, nearly 1 year after the COVID-19 outbreak in Bangladesh [20]. Bangladesh has a population of around 169 million with an area of 56,977 sq. miles, or 147,570 sq. km [28]. Islam is the most popular religion, with 90% of the population being Muslim. The population density of the country is 1140 per sq. km., and per capita income is 2824 USD (2021–2022). The main economic activity is agriculture [29].

### Sample size calculation

The sample size was calculated using the following Eq. (1):

1 $${\displaystyle \begin{array}{c}n=\frac{z^2 pq}{d^2}\\ {}\Rightarrow n=\frac{1.96^2\times 0.373\times \left(1-0.373\right)}{0.05^2}\\ {}\Rightarrow n=359.38\approx 359\end{array}}$$

Here,

*n* = number of samples

*z* = 1.96 (95% confidence level)

*p* = prevalence estimate (37.3% or 0.373)

*q* = (1-*p*)

*d* = precision limit or proportion of sampling error (0.05)

As a previous study reported 37.3% anxiety among Bangladeshi people early in the outbreak [24], a sample size of 503 participants was estimated assuming a 40% non-response rate. However, the present study’s sample size exceeded this estimate.

### Study procedure

Participants were recruited through an online survey using a convenience sampling technique. A semi-structured self-reported questionnaire (see Supplementary file) written in Bangla (the native language of participants) was designed and incorporated into the Google survey tool (Google Forms) for data collection. The link generated from the tool was shared across different social media platforms (e.g., Facebook, WhatsApp, etc.) and other online platforms (e.g., email, blogs, etc.) as an advertisement for participation in the survey. The questionnaire was pre-tested with 50 individuals before starting the final data collection for acceptability and clarity. Informed consent from each participant was obtained before starting the survey by asking, “*Are you willing to participate in this survey voluntarily?”* with “yes” or “no” responses*.* Those who responded positively were allowed to participate in this survey by giving access to the full questionnaire; otherwise, a blank survey form was submitted automatically. A total of 1033 participants completed the online survey form. Of these, 1004 participants were selected following quality control and manual check procedures to exclude incomplete and invalid surveys and were included in the final analysis. The inclusion criteria were i) being a Bangladeshi resident, ii) being aged ≥18 years, iii) good internet access, and iv) voluntary participation. The study protocol was reviewed and approved by the Biosafety, Biosecurity, and Ethical review board of the Jahangirnagar University, Savar, Dhaka-1342, Bangladesh [BBEC, JU/M 2020/COVID-19/(11)2]. The study was conducted following the Checklist for Reporting Results of Internet ESurveys (CHERRIES) guidelines [30].

### Measures

#### Socio-demographic measures

Participants’ socio-demographic information, including age, gender, marital status, education (below university/university), monthly family income, and residence (rural/urban), were collected during the survey. The respondents’ levels of education were primary (grades 1–5), secondary (grades 6–10), intermediate (grades 11–12), bachelor, master, and above, which were then further recoded into below university and university. Monthly family income was divided into four categories: less than 20,000 Bangladeshi Taka (BDT), 20,000–30,000 BDT, 30,000–40,000 BDT, and more than 40,000 BDT.

#### Lifestyle-related measures during COVID-19

Lifestyle-related measures were collected by asking questions about participants’ chronic health conditions (e.g., diabetes, high blood pressure, asthma/respiratory problems, heart disease, kidney problems, cancer, and any other conditions) (yes/no), self-rated health status (SRH) [31] (good/poor), worries due to COVID-19 (yes/no), experiencing prolonged home quarantine (yes/no), decreased household income due to COVID-19 (yes/no), unemployment of family members due to COVID-19 (yes/no), food scarcity (yes/no), sleep duration (< 7 hours/7–9 hours/> 9 hours), engaging in physical exercise (yes/no), average hours of browsing the internet, social media use (e.g., Facebook, WhatsApp) (not at all/rarely/sometimes/often/always), COVID-19 related news exposure (yes/no), tobacco smoking (yes/no), alcohol consumption (yes/no), and self-reported quality of life (SQL) [31] (good/poor). In terms of SRH and SQL, participants were asked, *“In general, how would you rate your overall health?*” and *“In general, how would you rate your overall quality of life?*”, respectively [31]. The responses were based on a five-point Likert scale with response options: “excellent”, “very good”, “good”, “fair”, and “poor”. In the present study, the responses for both SRH and SQL were recoded as good (excellent/very good/good) and poor (poor/fair). Average internet browsing hours were categorized as “< 2 hours”, “2–4 hours”, “4–6 hours”, and “> 6 hours”.

#### University of California, Los Angeles (UCLA) loneliness scale [32]

Participants’ loneliness was measured using the three-item translated Bangla version of the UCLA loneliness scale. Participants’ responses were based on a three-point Likert scale ranging from 1 (*hardly ever*) to 3 (*often*). The sum of the total scores ranged from 3 to 9, with higher scores indicating higher levels of loneliness. A score of ≥6 indicated loneliness, as in earlier research [12, 32–34]. The reliability or internal consistency of the measure was very good in the current sample (Cronbach’s α = 0.85).

#### Generalized anxiety disorder (GAD-7) [35]

The Bangla version of the seven-item GAD-7 was used for assessing the anxiety of the participants [36]. Participants’ responses were recorded using a four-point Likert scale ranging from 0 (*Not at all*) to 3 (*Nearly every day*). Total scores ranged from 0 to 21, with higher scores reflecting greater anxiety. A score of ≥10 indicated moderate to severe anxiety, as previously [12, 37, 38]. In the present study, the GAD-7 scale had excellent reliability (Cronbach’s alpha: α = 0.90).

#### Patient Health Questionnaire (PHQ-9) [39]

Participants’ depressive symptoms were measured using the Bangla version of the PHQ-9 [40]. Symptoms of depression during the past 2 weeks were recorded. Participant responses were recorded on a four-point Likert scale ranging from 0 (*Not at all*) to 3 (*Nearly every day*). Total scores ranged from 0 to 27, with higher scores reflecting greater depression [41, 42]. A score of ≥10 indicated depression, as previously [43, 44]. The internal consistency of the PHQ-9 scale in the present sample was very good (Cronbach’s α = 0.87).

#### Insomnia Severity Index (ISI) [45]

Participants’ insomnia was measured using the seven-item Bangla version of the ISI [46]. Each item is rated on a five-point Likert scale (e.g., 0 *“Not at all”* to 4 *“Extremely”*). Total scores ranged from 0 to 28, with higher scores reflecting greater insomnia severity. A final score ranging from 0 to 7 indicated no insomnia; a score of 8–14 indicated sub-threshold insomnia; a score of 15–21 indicated moderate insomnia; and a score of 22–28 indicated severe insomnia [14, 47]. A predetermined cutoff (ISI ≥ 10) was used to diagnose insomnia [47]. The Cronbach’s alpha was 0.91 in the present study.

#### Fear of COVID-19 Scale (FCV-19S) [48]

Participants’ COVID-19-related fear was measured using the seven-item Bangla version of the FCV-19S [49]. Participants’ responses were recorded using a five-point Likert scale ranging from 1 (*strongly disagree*) to 5 (*strongly agree*). Cumulative scores ranged from 7 to 35, with higher scores reflecting more COVID-19-related fear. The reliability or the internal consistency of the scale in the present study was very good (Cronbach’s α = 0.86).

### Statistical analysis

All statistical analyses were performed using two software packages (Microsoft Excel 2019 and SPSS version 25). Data cleaning, sorting, and coding were first performed using Microsoft Excel. Using SPSS, descriptive statistics (i.e., frequencies, percentages, means, and standard deviations) were computed. Inferential statistics involve conducting *t*-tests or one-way analyses of variance (ANOVAs) to determine the mean differences among variable groups. Bivariate Pearson correlation analyses were used for continuous variables. Variables that showed group differences/bivariate in initial analyses were included in hierarchical regression analyses. In each hierarchical regression model, socio-demographics were included in Block 1; Block 2 included lifestyle factors; and Block 3 included psychiatric symptoms. A *p*-value < 0.05 was considered significant for all statistical analyses. Exploratory analyses assessing whether insomnia, anxiety, and depression statistically mediated the relationship between loneliness and fear of COVID-19 were conducted with SPSS PROCESS and 5000 bootstrap resamples [50]. Loneliness was included as the independent variable, fear of COVID-19 as the dependent variable, and the severity of insomnia, anxiety, and depression as potential mediators. Associations, indirect effects, and bootstrapped confidence intervals (CIs) were calculated. Significant indirect effects were determined by CIs that did not include zero.

## Results

### General profile of participants

A total of 1004 individuals (mean age of 25.41 (SD = 7.80) years) comprised the study sample. Most participants were male (51.8%), unmarried (72.8%), had university educations (82.1%), and resided in urban areas (75.3%); many had monthly incomes between 30,000–40,000 BDT [319–426 US$] (27.5%) [1 US$ ≈ 93.91 BDT] (Table 1). Most denied chronic health conditions (81.6%) and reported good health status (60.6%). The distributions of continuous variables are presented in Table 2.

**Table 1:** Distributions of categorical variables by general psychiatric symptoms

Variables	Overall	Loneliness		Anxiety		Depression		Insomnia		Fear of COVID-19	
	n (%)	Mean (SD)	*t*	Mean (SD)	t	Mean (SD)	*t*	Mean (SD)	*t*	Mean (SD)	*t*
**Gender**											
Male	520 (51.8)	5.61 (1.78)	10.46**	6.99 (4.59)	2.52	9.91 (5.12)	1.66	9.43 (6.66)	0.75	17.5 (5.74)	3.83
Female	484 (48.2)	5.99 (1.86)		7.45 (4.63)		10.33 (5.23)		9.8 (6.72)		18.21 (5.63)	
**Marital status**											
Unmarried	731 (72.8)	5.85 (1.85)	7.29**	6.97 (4.57)	4.00*	9.87 (5.1)	8.33***	9.34 (6.64)	2.19	17.33 (5.39)	13.04***
Married	208 (20.7)	5.42 (1.7)		7.74 (4.67)		10.17 (4.96)		10.37 (7.18)		19.58 (5.83)	
In a relationship	65 (6.5)	6.31 (1.78)		8.25 (4.75)		12.58 (6.07)		10.2 (5.28)		18.11 (7.31)	
**Education**											
Below university	180 (17.9)	5.87 (1.76)	0.36	7.84 (4.38)	4.07*	11.17 (4.5)	9.31**	9.53 (6.47)	0.03	18.26 (5.94)	1.15
University	824 (82.1)	5.78 (1.84)		7.07 (4.66)		9.88 (5.29)		9.63 (6.74)		17.75 (5.64)	
**Monthly household income (BDT)**											
< 20,000	216 (21.5)	5.73 (1.97)	1.63	7.16 (5.02)	0.31	10.26 (5.21)	0.18	7.86 (6.18)	7.18***	17.18 (5.57)	3.59*
20,000–30,000	274 (27.3)	5.7 (1.74)		7.01 (4.18)		9.93 (4.82)		9.61 (6.8)		18.58 (5.94)	
30,000–40,000	276 (27.5)	5.73 (1.72)		7.34 (4.41)		10.14 (4.95)		10.22 (6.58)		18.13 (5.55)	
> 40,000	238 (23.7)	6.02 (1.91)		7.33 (4.95)		10.14 (5.78)		10.49 (6.85)		17.26 (5.59)	
**Residence**											
Urban	756 (75.3)	5.77 (1.82)	0.33	7.13 (4.66)	1.05	9.98 (5.23)	1.95	9.81 (6.67)	2.80	17.88 (5.56)	0.10
Rural	248 (24.7)	5.85 (1.87)		7.47 (4.47)		10.51 (4.98)		8.99 (6.71)		17.74 (6.09)	
**Chronic health conditions**											
Yes	185 (18.4)	6.25 (1.72)	14.60***	8.66 (4.61)	23.00***	11.52 (5.05)	17.06***	12.25 (6.33)	36.66***	18.5 (5.73)	3.00
No	819 (81.6)	5.69 (1.84)		6.88 (4.56)		9.79 (5.15)		9.01 (6.62)		17.69 (5.68)	
**Self-reported health status**											
Good	608 (60.6)	5.53 (1.78)	32.06***	6.6 (4.37)	27.88***	9.71 (5.08)	9.21**	8.8 (6.41)	23.22***	17.61 (5.58)	2.61
Poor	396 (39.4)	6.19 (1.83)		8.15 (4.82)		10.72 (5.26)		10.85 (6.92)		18.2 (5.85)	
**Worried due to COVID-19**											
Yes	697 (69.4)	5.85 (1.68)	1.86	7.56 (4.42)	13.26***	10.18 (4.8)	0.39	10.36 (6.55)	29.36***	19.05 (5.44)	113.48***
No	307 (30.6)	5.67 (2.13)		6.42 (4.95)		9.96 (5.94)		7.91 (6.68)		15.11 (5.3)	
**Experienced prolonged home quarantine**											
Yes	801 (79.8)	5.74 (1.87)	3.43	7.2 (4.71)	0.02	10.12 (5.18)	0.02	9.21 (6.77)	14.31***	17.84 (5.64)	< 0.01
No	203 (20.2)	6 (1.64)		7.26 (4.21)		10.07 (5.19)		11.18 (6.11)		17.85 (5.91)	
**Decreased household income due to COVID-19**											
Yes	644 (64.1)	5.83 (1.84)	0.84	7.5 (4.59)	7.07**	10.44 (5.08)	7.37**	9.78 (6.82)	1.23	18.15 (5.68)	5.22*
No	360 (35.9)	5.72 (1.81)		6.69 (4.62)		9.52 (5.31)		9.29 (6.44)		17.29 (5.69)	
**Unemployment of family members due to COVID-19**											
Yes	366 (36.5)	5.87 (1.74)	0.99	8.01 (4.42)	17.34***	10.81 (5.06)	10.50**	10.88 (7.07)	21.28***	18.48 (5.93)	7.16**
No	638 (63.5)	5.75 (1.88)		6.76 (4.66)		9.71 (5.2)		8.88 (6.34)		17.48 (5.53)	
**Food scarcity**											
Yes	387 (38.5)	5.85 (1.74)	.62	7.98 (4.35)	17.74***	10.54 (4.93)	4.30*	11 (7.02)	28.06***	19.1 (5.87)	31.55***
No	617 (61.5)	5.76 (1.88)		6.73 (4.71)		9.84 (5.31)		8.73 (6.31)		17.06 (5.44)	
**Average sleep duration**											
< 7 hours	304 (30.3)	5.66 (1.91)	1.98	7.38 (5.12)	0.30	9.49 (5.68)	3.19*	10.86 (7.12)	7.75***	18.19 (5.69)	3.41*
7–9 hours	532 (53.0)	5.8 (1.7)		7.12 (4.4)		10.41 (4.79)		9.04 (6.11)		17.97 (5.64)	
> 9 hours	168 (16.7)	6.01 (2.06)		7.2 (4.33)		10.28 (5.34)		9.13 (7.32)		16.82 (5.8)	
**Physical exercise**											
Yes	424 (42.2)	5.49 (1.87)	20.74***	6.85 (4.79)	4.42*	9.8 (5.23)	2.62	8.95 (6.96)	7.13**	17.64 (5.39)	0.89
No	580 (57.8)	6.02 (1.77)		7.47 (4.47)		10.34 (5.13)		10.09 (6.44)		17.99 (5.91)	
**Internet browsing hours**											
< 2 hours	123 (12.3)	5.02 (1.75)	14.85***	6.64 (4.68)	1.74	9.33 (4.89)	3.08*	7.47 (6.88)	7.95***	18.36 (5.55)	2.98*
2–4 hours	258 (25.7)	5.5 (1.65)		6.88 (4.47)		9.58 (4.76)		8.92 (6.02)		18.04 (5.48)	
4–6 hours	318 (31.7)	6.11 (1.68)		7.53 (4.51)		10.6 (4.88)		10.59 (6.31)		18.25 (5.9)	
> 6 hours	305 (30.4)	6.02 (2.03)		7.39 (4.8)		10.36 (5.83)		10.03 (7.27)		17.05 (5.65)	
**Social media use**											
Not at all	55 (5.5)	5.09 (1.92)	6.83***	8.45 (4.8)	5.15***	10.64 (4.63)	1.51	8.11 (7.7)	3.94**	18.96 (6.27)	2.61*
Rarely	86 (8.6)	5.8 (1.51)		8.1 (4.71)		10.57 (5.6)		10.19 (6.48)		18.81 (5.43)	
Sometime	285 (28.4)	5.58 (1.65)		6.62 (4.21)		9.53 (4.58)		9.57 (5.96)		18.25 (5.63)	
Often	374 (37.3)	5.79 (1.8)		6.84 (4.51)		10.17 (5.1)		8.97 (6.23)		17.54 (5.4)	
Always	204 (20.3)	6.28 (2.1)		8 (5.05)		10.49 (5.96)		10.99 (7.94)		17.12 (6.14)	
**COVID-19-related news exposure**											
Yes	641 (63.8)	5.74 (1.81)	1.69	7.26 (4.64)	0.23	9.93 (5.01)	2.09	9.39 (6.75)	1.83	18.41 (5.61)	18.02***
No	363 (36.2)	5.89 (1.86)		7.12 (4.57)		10.42 (5.44)		9.99 (6.56)		16.84 (5.72)	
**Tobacco smoking**											
Yes	186 (18.5)	5.89 (1.82)	0.61	7.67 (4.42)	2.28	10.38 (5.56)	0.60	10.72 (6.7)	6.36*	17.68 (5.6)	0.18
No	818 (81.5)	5.77 (1.83)		7.11 (4.65)		10.05 (5.08)		9.35 (6.66)		17.88 (5.72)	
**Alcohol consumption**											
Yes	73 (7.3)	6.49 (1.93)	11.65**	7.4 (4.66)	0.13	10.29 (5.96)	0.09	12.53 (7.25)	15.30***	17.41 (6.68)	0.45
No	931 (92.7)	5.74 (1.81)		7.2 (4.61)		10.1 (5.11)		9.38 (6.59)		17.88 (5.61)	
**Self-reported quality of life**											
Good	653 (65.0)	5.53 (1.75)	39.59***	6.74 (4.39)	20.26***	9.87 (5.12)	4.11*	9.01 (6.31)	15.18***	17.66 (5.58)	1.99
Poor	351 (35.0)	6.28 (1.88)		8.1 (4.89)		10.56 (5.25)		10.72 (7.21)		18.19 (5.89)	

*SD* Standard deviation, *BDT* Bangladeshi Taka

*p < 0.05, **p < 0.01, ***p < 0.001

**Table 2:** Distributions of all continuous variables including general psychiatric symptoms

Variables	α	Mean (SD)	Range	1	2	3	4	5
1. Age (years)	—	25.41 (7.80)	(18–60)	—				
2. Loneliness	0.85	5.79 (1.83)	(3–9)	−0.04	—			
3. Fear of COVID-19	0.86	17.84 (5.69)	(7–35)	0.19^**^	0.11^*^	—		
4. Anxiety	0.90	7.21 (4.62)	(0–21)	0.13^**^	0.42^**^	0.31^**^	—	
5. Depression	0.87	10.11 (5.18)	(0–27)	0.05	0.32^**^	0.23^**^	0.62^**^	—
6. Insomnia	0.91	9.61 (6.69)	(0–24)	0.10^*^	0.39^**^	0.26^**^	0.40^**^	0.27^**^

α Cronbach alpha, *SD* Standard deviation

*p < 0.01**p < 0.001

### Lifestyle during the COVID-19 pandemic

Most respondents reported worries due to COVID-19 infection (69.4%) and were in prolonged home quarantine (79.8%) (Table 1). Substantial proportions reported decreased household incomes (64.1%), joblessness of family members (36.5%), and food scarcity (38.5%) due to the pandemic. More than half (53.0%) reported they slept in a normal range (7–9 hours/day), and 57.8% reported not engaging in physical exercise during the COVID-19 pandemic. Participants reported considerable online behaviors during the COVID-19 pandemic, including internet use (> 6 hours/day: 30.4%) and social media use (e.g., Facebook, WhatsApp; most of the time: 20.3%). A sizeable minority reported smoking cigarettes (18.5%) and drinking alcohol (7.3%) during the pandemic. Most (63.8%) participants were exposed to regular COVID-19-related news. Finally, 35% reported a poor quality of life during the pandemic.

### Loneliness

The prevalence estimate of loneliness was 63.5%. Loneliness was positively associated with female gender, physical inactivity, frequent internet, and Facebook use, alcohol consumption, poor quality of life, anxiety, depression, and insomnia (Table 3). The regression model predicted 30% of the variance in loneliness [*F*_(13,990)_ = 33.36; *p* < .001].

**Table 3:** Hierarchical regression analysis predicting loneliness

Variables	***Model 1***				***Model 2***				***Model 3***					
	B	SE	β	***t***	B	SE	β	***t***	B	SE	β	***t***	ΔR^**2**^	R^**2**^_**Adj**_
**Block 1 – Socio-demographics** (*F*_(4,999)_ = 14.00; *p* < 0.001)													0.05	0.05
Gender^a^	0.36	0.11	0.10	3.17**	0.33	0.11	0.09	3.02**	0.28	0.10	0.08	2.83**		
Marital status^b^	−0.06	0.10	−0.02	− 0.66	− 0.02	0.09	− 0.01	− 0.21	− 0.13	0.08	− 0.04	−1.57		
Chronic health conditions^c^	− 0.55	0.15	− 0.12	−3.73***	− 0.66	0.15	− 0.14	−4.48***	− 0.24	0.14	− 0.05	−1.77		
Self-reported health status^d^	0.62	0.12	0.17	5.36***	0.22	0.13	0.06	1.72	0.04	0.12	0.01	0.33		
**Block 2 – Lifestyle factors** (*F*_(9,994)_ = 17.57; *p* < 0.001)													0.08	0.13
Physical exercise^c^					0.52	0.11	0.14	4.70***	0.40	0.10	0.11	3.96***		
Internet browsing hours^d^					0.27	0.07	0.15	4.04***	0.15	0.06	0.08	2.51*		
Social media use^e^					0.15	0.06	0.09	2.42*	0.15	0.06	0.09	2.64**		
Alcohol consumption^c^					−0.75	0.21	−0.11	−3.49**	−0.65	0.19	−0.09	−3.32**		
Self-reported quality of life^f^					0.59	0.13	0.15	4.46***	0.44	0.12	0.12	3.70***		
**Block 3 – Psychiatric symptoms** (*F*_(13,990)_ = 33.36; *p* < 0.001)													0.17	0.30
Fear of COVID-19									−0.01	0.01	−0.04	−1.32		
Anxiety									0.10	0.01	0.25	6.96***		
Depression									0.03	0.01	0.09	2.60*		
Insomnia									0.06	0.01	0.21	6.97***		

*B* Unstandardized regression coefficient, *SE* Standard error, *β* Standardized regression coefficient; ^a^1 = Male, 2 = Female; ^b^1 = Unmarried, 2 = Married, 3 = In a relationship; ^c^1 = Yes, 2 = No; ^d^1 = < 2 hours, 2 = 2–4 hours, 3 = 4–6 hours, 4 = > 6 hours; ^e^1 = Not at all, 2 = Rarely, 3 = Sometime, 4 = Often, 5 = Always; ^f^1 = Good, 2 = Poor. *p < 0.05, **p < 0.01, ***p < 0.001

### Anxiety

The prevalence estimate of moderate to severe anxiety was 26.3%. Anxiety was positively associated with older age, loneliness, depression, insomnia, and fear of COVID-19 (Table 4). Overall, the regression model predicted 49% of the variance in anxiety [*F*_(16,987)_ = 61.52; *p* < 0.001].

**Table 4:** Hierarchical regression analysis predicting anxiety

Variables	***Model 1***				***Model 2***				***Model 3***					
	B	SE	β	***t***	B	SE	β	***t***	B	SE	β	***t***	ΔR^**2**^	R^**2**^_**Adj**_
**Block 1 - Socio-demographics** (*F*_(5,998)_ = 12.49; *p* < 0.001)													0.06	0.05
Age	0.05	0.02	0.08	2.25*	0.03	0.02	0.05	1.23	0.04	0.02	0.07	2.38*		
Marital status^a^	0.34	0.26	0.04	1.28	0.31	0.26	0.04	1.19	−0.11	0.20	−0.02	− 0.58		
Education^b^	−0.21	0.39	−0.02	− 0.54	− 0.24	0.40	− 0.02	− 0.60	0.20	0.29	0.02	0.66		
Chronic health conditions^c^	−1.50	0.37	−0.13	−4.04***	− 1.63	0.38	− 0.14	−4.33***	− 0.16	0.29	− 0.01	− 0.55		
Self-reported health status^d^	1.43	0.29	0.15	4.91***	0.91	0.34	0.10	2.70**	0.37	0.25	0.04	1.45		
**Block 2 – Lifestyle factors** (*F*_(12,991)_ = 7.53; *p* < 0.001)													0.03	0.07
Worry due to COVID-19^c^					−0.80	0.32	−0.08	−2.50*	− 0.16	0.25	− 0.02	− 0.65		
Decreased household income due to COVID-19^c^					−0.18	0.33	−0.02	−0.55	0.06	0.25	0.01	0.25		
Unemployment of family members due to COVID-19^c^					−0.49	0.35	−0.05	−1.41	− 0.10	0.26	− 0.01	−0.38		
Food scarcity^c^					−0.40	0.37	−0.04	−1.07	− 0.18	0.28	− 0.02	−0.65		
Physical exercise^c^					0.59	0.29	0.06	1.99*	−0.07	0.22	− 0.01	− 0.30		
Social media use^d^					0.27	0.14	0.06	1.87	−0.09	0.11	− 0.02	−0.80		
Self-reported quality of life^e^					0.67	0.35	0.07	1.93	0.25	0.26	0.03	0.97		
**Block 3 – Psychiatric symptoms** (*F*_(16,987)_ = 61.52; *p* < 0.001)													0.42	0.49
Loneliness									0.47	0.07	0.19	7.10***		
Fear of COVID-19									0.10	0.02	0.12	4.66***		
Depression									0.44	0.02	0.49	19.55***		
Insomnia									0.10	0.02	0.15	5.51***		

*B* Unstandardized regression coefficient, *SE* Standard error, *β* Standardized regression coefficient; ^a^1 = Unmarried, 2 = Married, 3 = In a relationship; ^b^1 = Below university, 2 = University; ^c^1 = Yes, 2 = No; ^d^1 = Not at all, 2 = Rarely, 3 = Sometime, 4 = Often, 5 = Always; ^e^1 = Good, 2 = Poor. *p < 0.05, **p < 0.01, ***p < 0.001

### Depression

The prevalence estimate of moderate to severe depression was 46.4%. Depression was positively associated with decreased household income due to COVID-19, presence of food scarcity, more sleep, loneliness, and anxiety (Table 5). The regression model predicted 49% of the variance in depression [*F*_(14,989)_ = 49.31; *p* < 0.001].

**Table 5:** Hierarchical regression analysis predicting depression

Variables	***Model 1***				***Model 2***				***Model 3***					
	B	SE	β	***t***	B	SE	β	***t***	B	SE	β	***t***	ΔR^**2**^	R^**2**^_**Adj**_
**Block 1 - Socio-demographics** (*F*_(4,999)_ = 9.99; *p* < 0.001)													0.04	0.04
Marital status^a^	0.73	0.28	0.08	2.64**	0.72	0.28	0.08	2.55*	0.37	0.23	0.04	1.64		
Education^b^	−0.89	0.43	−0.07	−2.06*	−0.92	0.43	−0.07	−2.13*	−0.64	0.34	−0.05	−1.86		
Chronic health conditions^c^	−1.51	0.42	−0.11	−3.61***	−1.66	0.42	−0.12	−3.96***	− 0.41	0.34	− 0.03	− 1.19		
Self-reported health status^d^	0.93	0.33	0.09	2.83**	0.67	0.38	0.06	1.76	0.00	0.31	0.00	0.00		
**Block 2 – Lifestyle factors** (*F*_(10,993)_ = 6.39; *p* < 0.001)													0.02	0.05
Decreased household income due to COVID-19^c^					− 0.76	0.38	−0.07	−2.01*	− 0.63	0.30	− 0.06	− 2.10*		
Unemployment of family members due to COVID-19^c^					−0.59	0.39	−0.06	−1.52	−0.24	0.31	−0.02	− 0.78		
Food scarcity^c^					0.15	0.41	0.01	0.38	0.69	0.33	0.07	2.11*		
Average sleep duration^d^					0.25	0.24	0.03	1.06	0.47	0.19	0.06	2.40*		
Internet browsing hours^e^					0.59	0.16	0.11	3.60***	0.21	0.13	0.04	1.54		
Self-reported quality of life^f^					0.19	0.39	0.02	0.49	−0.39	0.31	−0.04	−1.25		
**Block 3 – Psychiatric symptoms** (*F*_(14,989)_ = 49.31; *p* < 0.001)													0.35	0.40
Loneliness									0.19	0.08	0.07	2.29*		
Fear of COVID-19									0.04	0.02	0.05	1.74		
Anxiety									0.64	0.03	0.57	19.48***		
Insomnia									0.01	0.02	0.01	0.27		

*B* Unstandardized regression coefficient, *SE* Standard error, *β* Standardized regression coefficient; ^a^1 = Unmarried, 2 = Married, 3 = In a relationship; ^b^1 = Below university, 2 = University; ^c^1 = Yes, 2 = No; ^d^1 = < 7 hours, 2 = 7–9 hours, 3 = > 9 hours; ^e^1 = < 2 hours, 2 = 2–4 hours, 3 = 4–6 hours, 4 = > 6 hours; ^f^1 = Good, 2 = Poor. *p < 0.05, **p < 0.01, ***p < 0.001

### Insomnia

The prevalence estimate of insomnia was 50.7%. Among those with insomnia, estimates of subthreshold, moderate, and severe insomnia were 37.8, 17.1, and 4.7%, respectively. Insomnia was positively associated with higher monthly income, chronic health conditions, worries regarding COVID-19 infection, not having prolonged home quarantine, joblessness of family members due to COVID-19, food scarcity, less sleep, alcohol consumption, loneliness, anxiety, and fear of COVID-19 (Table 6). The regression model predicted 32% of the variance in insomnia [*F*_(19,984)_ = 26.22; *p* < 0.001].

**Table 6:** Hierarchical regression analysis predicting insomnia

Variables	***Model 1***				***Model 2***				***Model 3***					
	B	SE	β	***t***	B	SE	β	***t***	B	SE	β	***t***	ΔR^**2**^	R^**2**^_**Adj**_
**Block 1 - Socio-demographics** (*F*_(4,999)_ = 21.45; *p* < 0.001)													0.08	0.08
Age		0.03	0.08	2.44*	0.02	0.03	0.03	0.77	0.01	0.03	0.01	0.36		
Monthly household income^a^		0.19	0.14	4.61***	0.86	0.19	0.14	4.51***	0.80	0.17	0.13	4.60***		
Chronic health conditions^b^		0.53	−0.17	−5.42***	−3.02	0.52	−0.18	−5.78***	−1.94	0.48	−0.11	−4.02***		
Self-reported health status^c^		0.42	0.14	4.64***	0.92	0.46	0.07	2.00*	0.44	0.42	0.03	1.05		
**Block 2 – Lifestyle factors** (*F*_(15,988)_ = 16.52; *p* < 0.001)													0.12	0.19
Worry due to COVID-19^b^					−2.07	0.44	−0.14	−4.69***	−1.16	0.42	−0.08	−2.74**		
Prolonged home quarantine^b^					2.35	0.50	0.14	4.70***	1.98	0.46	0.12	4.32***		
Unemployment of family members due to COVID-19^b^					−1.44	0.47	−0.10	−3.05**	−1.19	0.43	−0.09	−2.75**		
Food scarcity^b^					−1.49	0.48	−0.11	−3.11**	−1.17	0.44	−0.09	−2.69**		
Average sleep duration^c^					−1.59	0.29	−0.16	−5.52***	−1.44	0.27	−0.15	−5.44***		
Physical exercise^b^					1.31	0.40	0.10	3.31**	0.66	0.37	0.05	1.81		
Internet browsing hours^d^					0.78	0.24	0.12	3.20**	0.41	0.22	0.06	1.83		
Social media use^e^					0.49	0.23	0.08	2.15*	0.40	0.21	0.06	1.92		
Tobacco smoking^b^					0.36	0.53	0.02	0.67	0.11	0.48	0.01	0.22		
Alcohol consumption^b^					−1.99	0.81	−0.08	−2.46*	−1.59	0.74	−0.06	−2.14*		
Self-reported quality of life^f^					1.30	0.47	0.09	2.75**	0.53	0.44	0.04	1.21		
**Block 3 – Psychiatric symptoms** (*F*_(19,984)_ = 26.22; *p* < 0.001)													0.14	0.32
Loneliness									0.79	0.11	0.22	7.11***		
Fear of COVID-19									0.13	0.03	0.11	3.85***		
Anxiety									0.27	0.05	0.19	5.18***		
Depression									0.03	0.04	0.02	0.70		

*B* Unstandardized regression coefficient, *SE* Standard error, *β* Standardized regression coefficient; ^a^1 = < 20,000, 2 = 20,000-30,000, 3 = 30,000-40,000, 4 = > 40,000; ^b^1 = Yes, 2 = No; ^c^1 = < 7 hours, 2 = 7–9 hours, 3 = > 9 hours; ^d^1 = < 2 hours, 2 = 2–4 hours, 3 = 4–6 hours, 4 = > 6 hours; ^e^1 = Not at all, 2 = Rarely, 3 = Sometime, 4 = Often, 5 = Always; ^f^1 = Good, 2 = Poor. *p < 0.05, **p < 0.01, ***p < 0.001

### Fear of COVID-19

The mean FCV-19S score was 17.84 (SD = 5.69). Fear of COVID-19 was positively associated with older age, COVID-19-related worries, and employment of family members, COVID-19 news exposure, anxiety, depression, and insomnia (Table 7). The regression model predicted 22% of the variance in fear of COVID-19 [*F*_(15,988)_ = 20.12; *p* < 0.001].

**Table 7:** Hierarchical regression analysis predicting fear of COVID-19

Variables	***Model 1***				***Model 2***				***Model 3***					
	B	SE	β	***t***	B	SE	β	***t***	B	SE	β	***t***	ΔR^**2**^	R^**2**^_**Adj**_
**Block 1 - Socio-demographics** (*F*_(3,1000)_ = 13.01; *p* < 0.001)													0.04	0.04
Age	0.12	0.03	0.17	4.99***	0.08	0.03	0.11	3.25**	0.07	0.02	0.09	2.66**		
Marital status^a^	0.46	0.33	0.05	1.41	0.60	0.31	0.06	1.93	0.43	0.30	0.05	1.45		
Monthly household income^b^	0.01	0.17	0.00	0.04	−0.02	0.17	0.00	−0.13	−0.14	0.16	−0.03	−0.86		
**Block 2 – Lifestyle factors** (*F*_(11,992)_ = 15.86; *p* < 0.001)													0.12	0.14
Worry due to COVID-19^c^					−3.37	0.39	−0.27	−8.74***	−3.04	0.37	−0.25	−8.22***		
Decreased household income due to COVID-19^c^					0.21	0.40	0.02	0.53	0.27	0.38	0.02	0.72		
Unemployment of family members due to COVID-19^c^					0.56	0.41	0.05	1.36	0.94	0.39	0.08	2.39*		
Food scarcity^c^					−0.99	0.44	−0.09	−2.26*	−0.74	0.42	−0.06	−1.75		
Average sleep duration^d^					−0.64	0.25	−0.08	−2.53*	− 0.44	0.25	− 0.05	−1.80		
Internet browsing hours^e^					0.13	0.21	0.02	0.61	−0.11	0.20	−0.02	− 0.55		
Social media use^f^					−0.22	0.20	−0.04	−1.12	− 0.20	0.19	− 0.04	− 1.07		
COVID-19 news exposure^c^					−1.46	0.36	−0.12	−4.05***	−1.51	0.34	−0.13	−4.41***		
**Block 3 – Psychiatric symptoms** (*F*_(15,988)_ = 20.12; *p* < 0.001)													0.08	0.22
Loneliness									−0.09	0.10	−0.03	− 0.91		
Anxiety									0.21	0.05	0.17	4.26***		
Depression									0.11	0.04	0.10	2.73**		
Insomnia									0.11	0.03	0.13	4.00***		

*B* Unstandardized regression coefficient, *SE* Standard error, *β* Standardized regression coefficient; ^a^1 = Unmarried, 2 = Married, 3 = In a relationship; ^b^1 = < 20,000, 2 = 20,000-30,000, 3 = 30,000-40,000, 4 = > 40,000; ^c^1 = Yes, 2 = No; ^d^1 = < 7 hours, 2 = 7–9 hours, 3 = > 9 hours; ^e^1 = < 2 hours, 2 = 2–4 hours, 3 = 4–6 hours, 4 = > 6 hours; ^f^1 = Not at all, 2 = Rarely, 3 = Sometime, 4 = Often, 5 = Always. *p < 0.05, **p < 0.01, ***p < 0.001

**Fig. 1:**
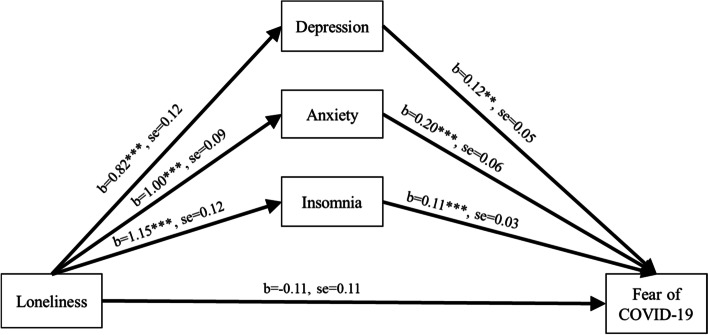
Depression, anxiety, and insomnia mediate the relationship between loneliness and fear of COVID-19. Note: Results of mediation analysis depicting coefficients, standard error (se), and significance of associations. Mediation analysis controlled for socio-demographics and lifestyle factors. *p ≤ 0.05, **p ≤ 0.01, ***p ≤ 0.001. The significant relationship between loneliness and fear of COVID-19 was no longer significant in the mediation model outlined above, indicating that insomnia, anxiety and depression mediated the relationship between loneliness and fear of COVID-19

Figure 1 depicts the path coefficients of the indirect relationships between loneliness and fear of COVID-19 through insomnia, anxiety, and depression. The model variables accounted for significant variance in fear of COVID-19 [*R*^2^ = 0.25, *F*_(25, 978)_ = 14.71, *p* < 0.001]. Loneliness was associated with greater levels of insomnia, anxiety, and depression (all *p* < 0.001), which in turn, were associated with greater fear of COVID-19 at the *p* < 0.001 level, except for depression (*p* = 0.01). Loneliness had significant indirect effects on fear of COVID-19 through insomnia (b = 0.13, se = 0.04, CI = 0.06–0.21), anxiety (b = 0.20, se = 0.05, CI = 0.09–0.31), and depression (b = 0.10, se = 0.04, CI = 0.03–0.18). Additionally, the association between loneliness and fear of COVID-19 (b = 0.32, se = 0.10, *p* < 0.001) reduced to non-significance upon adding mediating variables (b = − 0.11, se = 0.11, *p* = 0.30). Hence, insomnia, anxiety, and depression mediated the relationship between loneliness and fear of COVID-19.

## Discussion

The COVID-19 pandemic has been deemed by some as the world’s most disastrous crisis, and considerable psychological concerns have been reported among people of all ages [51–53]. Investigation into the psychological burdens due to this pandemic (with respect to psychiatric disorders and less severe manifestations) is important in considering interventions to promote mental health and well-being [54]. The present study investigated the prevalence of psychiatric symptomatology and associated factors among Bangladeshi individuals approximately 1 year following the COVID-19 outbreak. We observed high levels of loneliness, depression, anxiety, insomnia, and fear of COVID-19 that were interrelated and associated with specific health correlates.

The prevalence estimate of loneliness was 63.5% in the present study, which indicates a higher prevalence of loneliness among Bangladeshi general people, and this is consistent with a previous study that also found a higher prevalence of loneliness (71%) among Bangladeshi general people during this COVID-19 pandemic [55]. This is plausible due to the fact that social isolation during the COVID-19 pandemic is a risk factor for loneliness according to a previous study [56]. However, similar surveys during the COVID-19 pandemic among the citizens of Canada [57] and the United Kingdom [12] found prevalence estimates of loneliness of 34.8% and 27%, respectively. This discrepancy may be related to the methods used in assessing the loneliness symptoms. Loneliness was more prevalent in women than in men in the current study, consistent with findings from other countries during the COVID-19 pandemic [58, 59]. Women with fewer integrated, cohesive groups of friends have reported feeling more lonely than men [60], and gender-related factors promoting loneliness during the COVID-19 warrant more study. Loneliness associated with COVID-19 has previously been linked to higher levels of psychological distress and poorer quality of life [61], consistent with the present findings. Loneliness was also associated with frequent internet use, similar to findings among Italian adults [43]. Loneliness was also found to be linked to the high-frequency use of social media (e.g., Facebook) in the present study, similar to findings from other investigations [62]. Loneliness is also connected to psychiatric symptomatology including anxiety and depression. Increased loneliness resulting from spatial distancing efforts during the COVID-19 pandemic may constitute a serious concern that may contribute to increases in depression and anxiety [63, 64], and this possibility warrants additional investigation.

The prevalence of moderate to severe anxiety was 26.3%. Banna et al. found that the prevalence of anxiety symptoms among Bangladeshi adults during an earlier stage of the COVID-19 pandemic was 33.7% [15]. In the present study, anxiety symptoms were particularly prevalent among older individuals. Similar findings were found in prior COVID-19 research in Bangladesh [15, 24]. This finding may be attributed to anxiety regarding the higher COVID-19 death rate among older people [24]. However, other data suggest that older adults may be more resilient to anxiety, depression, and stress-related mental health disorders [65, 66]. Anxiety was also associated with loneliness in the current study. In line with previous findings, loneliness due to social distancing during the COVID-19 pandemic was the main factor linked to depression and anxiety [63]. The results underscore that loneliness during pandemic situations may have a notable psychiatric impact. Accordingly, policymakers and mental health practitioners should emphasize the importance of safe social interactions while remaining connected [67]. Moreover, anxiety was linked to insomnia, in line with previous studies [68, 69]. In Italy, anxiety related to COVID-19 was associated with disturbed sleep [70]. Moreover, a study conducted among Swedish general people that suggests a bidirectional relationship between anxiety (and depression) and insomnia [71].

The present study found slightly less than half of the participants (46.4%) were suffering from moderate to severe depression. A prior Bangladeshi study found a considerably higher percentage earlier in the pandemic [15]. Presently, depression is associated with decreased household income due to COVID-19. Lower levels of household income have been associated with psychological distress [72]. Consistent with the current findings, food insecurity during the COVID-19 pandemic has been linked to mental health issues such as anxiety and depression, and the impact of food insecurity has been estimated to be three times that of joblessness during the pandemic [73]. Excessive sleep during the COVID-19 pandemic was also associated with depression. This finding aligns with a prior Bangladeshi longitudinal study [36] and other findings [74] but appears to contrast with another prior Bangladeshi report [75] that observed no association between hours of sleep and depression. Loneliness was also significantly associated with depression. A recent review suggests loneliness has been elevated during the COVID-19 pandemic, with 43% of respondents scoring above validated thresholds, and has been strongly associated with depression and suicidal ideation [64].

In the current study, prevalence estimates of subthreshold, moderate, and severe insomnia were 37.8%, 17.1%, and 4.7%, respectively, which are similar to those from a prior Bangladeshi study [14]. Consistent with other studies, this study found that participants with chronic conditions had elevated odds of insomnia [14, 76]. Insomnia has been linked to worries about COVID-19 infection previously in Greek and French populations [77, 78], and sleep patterns and quality may also relate to worries about contamination and financial concerns [77]. Insomnia in this study was linked with not having prolonged home quarantine. The COVID-19 situation, in which people were often compelled to perform work or study at home, with accompanying concerns about health risks and social isolation, has been associated with disrupted sleep patterns [79]. According to the findings of this study, participants with family members who lost jobs during the pandemic had higher odds of insomnia. Because losing a job often generates a great deal of insecurity in terms of earning a living, insomnia may develop [80]. Alcohol consumption is also paired with insomnia in this study. Aoyama et al. [81] found that alcohol intake was associated with insomnia. Not surprisingly, reduced sleep duration was associated with insomnia. While the emergence of sleep disturbances in response to stressful life events is to be expected, contrary findings exist. Li et al. [82] found an increase in total sleep time and a decrease in overall sleep quality among people during the COVID-19 pandemic. Loneliness was linked to insomnia among the current participants. Loneliness may induce increased feelings of vulnerability, thereby increasing arousal, and generating insomnia [78].

This study’s mean FCV-19S score was 17.84, comparable to a Bangladeshi study conducted earlier during the COVID-19 pandemic, which revealed a mean score of 18.53 [83]. In the current study, older people were more likely to be fearful of COVID-19. This is unsurprising given that older people are more susceptible to mortality and disability as a result of COVID-19 [84]. Consequently, they may be more fearful of contracting the disease [85, 86]. Participants in this study who were more worried about COVID-19 infection were more likely to be fearful of COVID-19. A prior study reported a high level of worry as being associated with increased anxiety and fear of COVID-19 [87]. In the current study, participants with family members still working during the pandemic were more fearful of contracting the virus. Since the majority of people who continued working during COVID-19 were more likely to go outside of the house, they may have been considered more prone to becoming infected with COVID-19 and spreading it to family members [88]. Additionally, participants who had been exposed to COVID-19 news on a regular basis were more likely to report higher levels of fear of COVID-19. This finding may reflect information and possible misinformation about COVID-19 having been spread through various media outlets, possibly generating widespread fear among the public [89]. The present study also found that anxiety and depression were associated with fear of COVID-19. Similarly, an Israeli study found that anxiety, stress, and depression were associated with fear of COVID-19 [90]. It is also evident that the presence of fear is associated with the occurrence of sleep problems and as such, the fear related to COVID-19 appears to be the same [14]. Stress may lead to insomnia and other psychological problems [91].

In the present study, anxiety, depression, and insomnia also mediated the relationship between loneliness and fear of COVID-19. The relationship between loneliness and fear of COVID-19 is consistent with prior reports [92, 93]. The correlations between loneliness and anxiety/depression/insomnia, and between anxiety/depression/insomnia and fear of COVID-19 have also been documented separately [61, 63, 64, 78, 90, 91]. However, the relationship between loneliness and fear of COVID-19, as mediated by anxiety, depression, and insomnia, is novel and suggests possible etiological factors that may be targeted in interventions aimed at reducing fear of COVID-19. The extent to which such pathways may extend to fears of other illnesses warrants additional direct examination, as does the investigated pathway in longitudinal data.

### Limitations

Study limitations warrant consideration. First, this study used a cross-sectional design; thus, causality cannot be established. Longitudinal studies would help in this regard. Second, responses were self-reported and may be subject to biases and tendencies to report socially desirable responses. Third, the use of online for surveying respondents is prone to selection bias due to the necessity of having internet access to participate. Finally, the sample was largely composed of single individuals with a high level of education and low medical comorbidity, so results might not generalize or represent an accurate reflection of the whole Bangladeshi population or groups from other jurisdictions.

## Conclusions

In summary, our findings suggest that the COVID-19 pandemic may be having longer-term psychological impacts on Bangladeshi people. Loneliness, anxiety, depression, and insomnia were frequently acknowledged, and considerable levels of fear of COVID-19 were observed. Although these domains appear correlated, specific socio-demographic and lifestyle-related factors were observed to link to specific aspects of mental health. One year into the COVID-19 pandemic, special attention may be needed to protect the mental health of potentially vulnerable populations including older adults, women, people with frequent internet users, and those who are worried about COVID-19 infection. Since a pandemic like COVID-19 has the potential to exacerbate social disparities in psychological health in subtle ways, authorities and health systems should consider developing national guidelines for dealing with psychological distress during and after the COVID-19 pandemic. Better preparedness for infectious disease outbreaks, including investment in psychiatric interventions and the use of validated instruments to assist in the identification and helping of at-risk individuals, may foster resilience and enhance public safety during events like the COVID-19 pandemic.

## Supplementary Information

**Additional file 1.** Questionnaire: General psychiatric symptoms among Bangladeshi people approximately 1 year after the onset of the COVID-19 pandemic.

## Data Availability

The datasets generated and/or analyzed during the current study are available from the corresponding author on reasonable request.

## Abbreviations

COVID-19: Coronavirus disease-2019
CHERRIES: Checklist for Reporting Results of Internet ESurveys
BDT: Bangladeshi Taka
SRH: Self-rated health status
SQL: Self-reported quality of life
UCLA: University of California, Los Angeles
GAD-7: Generalized Anxiety Disorder
PHQ-9: Patient Health Questionnaire
ISI: Insomnia Severity Index
FCV-19S: Fear of COVID-19 Scale
SPSS: Statistical Package for the Social Sciences
ANOVA: Analysis of variance
CIs: Confidence intervals
SD: Standard deviation
