# Supplementary material for: General psychiatric symptoms among Bangladeshi people approximately one year after the onset of the COVID-19 pandemic
Source: BMC Psychiatry. 2022 Sep 19;22:615. doi: 10.1186/s12888-022-04232-3 (PMC9483885; doi:10.1186/s12888-022-04232-3)
Supplement: Supplementary file 1 — Additional file 1. Questionnaire: General psychiatric symptoms among Bangladeshi people approximately 1 year after the onset of the COVID-19 pandemic. [file 12888_2022_4232_MOESM1_ESM.docx]

**Supplementary file**

**Questionnaire: General psychiatric symptoms among Bangladeshi people approximately one year after the onset of the COVID-19 pandemic**

**[Note: For each question mark only one oval]**

| Do you willing to participate in this study? |
| --- |
| 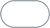 Yes |
| 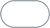 No |

| **Section: A**  **Socio-demographics** |
| --- |

| A1. What is your age? |
| --- |
| _________________years |
| A2. What is your gender? |
| 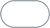 Male |
| 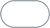 Female |
| A3. Marital status? |
| 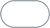 Unmarried |
| 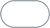 Married |
| 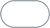 In a relationship |
| A4. What is your highest level of education that you have completed? |
| 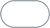 Primary (Grades 1-5) |
| 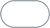 Secondary (Grades 6-10) |
| 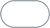 Intermediate (Grades 11-12) |
| 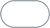 Bachelor |
| 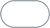 Master's and above |
| A5. What is your monthly family income (BDT)? |
| 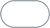 < 20000 BDT |
| 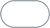 20000-30000 BDT |
| 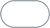 30000-40000 BDT |
| 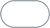 > 40000 BDT |
| A6. Which area do you currently live in? |
| 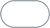 Urban |
| 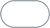 Rural |

| **Section: B**  **Health status, COVID-19 impact and Lifestyle related information** |
| --- |

| B1. Do you suffering from any chronic diseases such as diabetes, cardiovascular disease, cancer etc.? |
| --- |
| 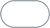 Yes |
| 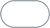 No |
| B2. How do you rate your overall health? |
| 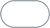 Excellent |
| 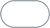 Very good |
| 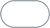 Good |
| 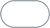 Fair |
| 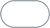 Poor |
| B3. Do you worried about COVID-19? |
| 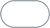 Yes |
| 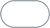 No |
| B4. Have you ever experienced prolonged home quarantine? |
| 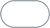 Yes |
| 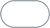 No |
| B5. Have your household income decreased due to COVID-19? |
| 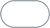 Yes |
| 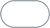 No |
| B6. Has anyone in your family become unemployed due to COVID-19? |
| 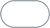 Yes |
| 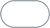 No |
| B7. Is your family suffering from food scarcity? |
| 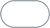 Yes |
| 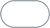 No |
| B8. How long do you currently sleep every night on average? |
| 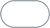 < 7 hours |
| 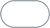 7-9 hours |
| 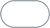 > 9 hours |
| B9. Do you involved in any type of physical exercise? |
| 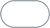 Yes |
| 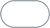 No |
| B10. How many hours a day do you currently spend on browsing the internet? |
| 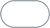 < 2 hours |
| 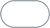 2-4 hours |
| 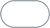 4-6 hours |
| 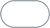 > 6 hours |
| B11. How do you rate your Social media use? |
| 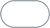 Not at all |
| 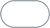 Rarely |
| 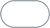 Sometime |
| 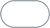 Often |
| B12. Do you exposed to COVID-19 related news regularly? |
| 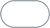 Yes |
| 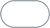 No |
| B13. Do you smoke tobacco? |
| 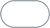 Yes |
| 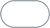 No |
| B14. Do you consume alcohol? |
| 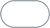 Yes |
| 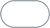 No |
| B15. How do you rate your overall quality of life? |
| 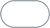 Excellent |
| 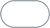 Very good |
| 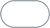 Good |
| 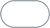 Fair |
| 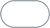 Poor |

| **Section: C**  **Psychiatric health related information** |
| --- |

**C1. Assessment of Loneliness**

| **Questions** *(The next questions are about how you feel about different aspects of your life. For each one, tell me how often you feel that way.)* | Hardly Ever | Some of the Time | Often |
| --- | --- | --- | --- |
| 1. How often do you feel that you lack companionship? | 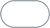 | 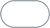 | 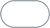 |
| 1. How often do you feel left out? | 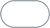 | 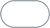 | 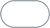 |
| 1. How often do you feel isolated from others? | 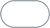 | 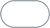 | 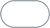 |

**C2. Assessment of Generalized Anxiety**

| **Questions** (*Over the last two weeks, how often have you been bothered by the following problems?)* | Not at all | Several days | More than half the days | Nearly every day |
| --- | --- | --- | --- | --- |
| 1. Feeling nervous, anxious or on edge | 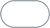 | 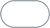 | 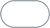 | 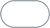 |
| 1. Not being able to stop or control worrying | 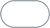 | 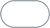 | 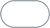 | 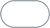 |
| 1. Worrying too much about different things | 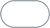 | 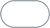 | 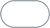 | 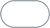 |
| 1. Trouble relaxing | 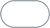 | 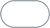 | 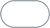 | 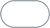 |
| 1. Being so restless that it is hard to sit still | 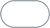 | 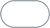 | 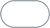 | 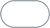 |
| 1. Becoming easily annoyed or irritable | 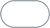 | 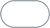 | 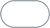 | 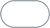 |
| 1. Feeling afraid as if something awful might happen | 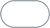 | 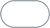 | 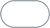 | 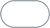 |

**C3. Assessment of Depression**

| **Questions** (*Over the last two weeks, how often have you been bothered by the following problems?)* | Not at all | Several days | More than half the days | Nearly every day |
| --- | --- | --- | --- | --- |
| 1. Little interest or pleasure in doing things | 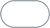 | 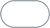 | 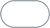 | 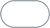 |
| 1. Feeling down, depressed or hopeless |  |  |  |  |
| 1. Trouble falling asleep, staying asleep, or sleeping too much |  |  |  |  |
| 1. Feeling tired or having little energy |  |  |  |  |
| 1. Poor appetite or overeating |  |  |  |  |
| 1. Feeling bad about yourself - or that you’re a failure or have let yourself or your family down |  |  |  |  |
| 1. Trouble concentrating on things, such as reading the newspaper or watching television |  |  |  |  |
| 1. Moving or speaking so slowly that other people could have noticed. Or, the opposite - being so fidgety or restless that you have been moving around a lot more than usual |  |  |  |  |
| 1. Thoughts that you would be better off dead or of hurting yourself in some way |  |  |  |  |

**C4. Assessment of Insomnia**

Please rate the current (i.e. last 2 weeks) severity of your insomnia problem

| **Questions** | | | **None** | **Mild** | | **Moderate** | **Severe** | | **Very severe** |
| --- | --- | --- | --- | --- | --- | --- | --- | --- | --- |
| 1. Difficulty falling asleep | | |  |  | |  |  | |  |
| 1. Difficulty staying asleep | | |  |  | |  |  | |  |
| 1. Problems waking up too early | | |  |  | |  |  | |  |
| 1. How SATISFIED/DISSATISFIED are you with your CURRENT sleep pattern? | | | | | | | | | |
| Very  Satisfied | Satisfied | Moderately  Satisfied | | | Dissatisfied | | | Very  Dissatisfied | |
|  |  |  | | |  | | |  | |
| 1. How NOTICEABLE to others do you think your sleep problem is in terms of impairing the quality of your life? | | | | | | | | | |
| Not at all  Noticeable | A Little | Somewhat | | | Much | | | Very Much Noticeable | |
|  |  |  | | |  | | |  | |
| 1. How WORRIED/DISTRESSED are you about your current sleep problem? | | | | | | | | | |
| Not at all  Worried | A Little | Somewhat | | | Much | | | Very Much Worried | |
|  |  |  | | |  | | |  | |
| 1. To what extent do you consider your sleep problem to INTERFERE with your daily functioning (e.g. daytime fatigue, mood, ability to function at work/daily chores, concentration, memory, mood, etc.) CURRENTLY? | | | | | | | | | |
| Not at all  Interfering | A Little | Somewhat | | | Much | | | Very Much Interfering | |
|  |  |  | | |  | | |  | |

**C5. Assessment of Fear of COVID-19**

| **Questions** | **Strongly disagree** | **Disagree** | **Neither agree nor disagree** | **Agree** | **Strongly agree** |
| --- | --- | --- | --- | --- | --- |
| 1. I am most afraid of coronavirus-19. |  |  |  |  |  |
| 1. It makes me uncomfortable to think about coronavirus-19. |  |  |  |  |  |
| 1. My hands become clammy when I think about coronavirus-19. |  |  |  |  |  |
| 1. I am afraid of losing my life because of coronavirus-19. |  |  |  |  |  |
| 1. When watching news and stories about coronavirus-19 on social media, I become nervous or anxious. |  |  |  |  |  |
| 1. I cannot sleep because I’m worrying about getting coronavirus-19 |  |  |  |  |  |
| 1. My heart races or palpitates when I think about getting coronavirus-19 |  |  |  |  |  |

**“Thank You”**
